# Message on a bottle: are alcohol warning labels about cancer appropriate?

**DOI:** 10.1186/s12889-016-2812-8

**Published:** 2016-02-11

**Authors:** Emma R. Miller, Imogen J. Ramsey, Genevieve Y. Baratiny, Ian N. Olver

**Affiliations:** Discipline of Public Health, Flinders University, GPO Box 2100, Bedford Park, 5001 South Australia; Mt Isa Centre for Rural and Remote Health, James Cook University, Mt Isa, Queensland; Samson Institute for Health Research, University of South Australia, Adelaide, South Australia

**Keywords:** Alcohol, Cancer prevention, Warning labels

## Abstract

**Background:**

Although most Australians are unaware of the risk, there is strong evidence for a direct link between alcohol consumption and many types of cancer. Warning labels on alcohol products have been proposed as a cost-effective strategy to inform the community of this health risk. We aimed to identify how Australians might respond to such an approach.

**Methods:**

We conducted a national online survey canvassing responses to four separate cancer warning messages on labels. The graphically presented messages were informed by qualitative data from a series of focus groups among self-identified ‘light-to-moderate’ drinkers. For each label, participants were asked their level of agreement with impact statements about raising awareness, prompting conversation, influencing drinking behaviour and educating others about cancer risk. We analysed responses according to demographic and other factors, including self-reported drinking behaviour (using the 3-item Alcohol Use Disorder Test – AUDIT-C – scores).

**Results:**

Approximately 1600 participants completed the survey, which was open to all Australian adults over a period of 1 month in 2014. Overall, the labels were well received, with the majority (>70 %) agreeing all labels could raise awareness and prompt conversations about the cancer risk associated with alcohol. Around 50 % or less agreed that the labels could influence drinking behaviour, but larger proportions agreed that the labels would prompt them to discuss the issue with family and friends. Although sex, AUDIT-C score and age were significantly associated with agreement on bivariate analysis, multivariate analyses demonstrated that being inclined to act upon warning label recommendations in general was the most important predictor of agreement with all of the impact statements. Having a low AUDIT-C score also predicted agreement that the labels might prompt behaviour change in friends.

**Conclusions:**

The findings suggest that providing detailed warnings about cancer risk on alcohol products is a viable means of increasing public awareness of the health risks associated with alcohol consumption. Further research is needed to explore the ability of such warnings to influence behavioural intentions and actual drinking behaviour.

## Background

Alcohol is the most widely used drug in Australia [1] and has been identified as a Class 1 carcinogen [2]. Drinking alcohol at any level increases the risk of developing an alcohol-related cancer [3]. There is evidence for a dose-response relationship between alcohol and cancer risk, such that the level of cancer risk increases proportionately to the level of alcohol consumption [4–6]. A comprehensive review of the scientific evidence by the World Cancer Research Fund and the American Institute for Cancer Research reported a causal relationship between alcohol consumption and development of a number of cancers, including cancers of the mouth, larynx, oesophagus, pharynx, bowel, breast and liver [3, 6]. It is estimated that around 5000 cases of cancer per year in Australia are attributable to long-term use of alcohol [6] and nearly 340 thousand deaths worldwide are the result of alcohol-attributable cancers [7].

Although there is growing awareness in the Australian population of the dangers of heavy episodic alcohol consumption and drinking during pregnancy, there is little understanding of the health risks associated with ‘light’ to ‘moderate’ alcohol consumption [8] which, in Australia, has been classified as drinking less than two standard drinks per day for men and women [9]. Surveys show that most Australian adults drink alcohol; about half do so at levels that increase their risk of alcohol-related harm in the short-term, and about one-quarter at levels that increase their risk of alcohol-related harm in the long-term [8]. The 2013 National Drug Strategy Household Survey [10] explored awareness of health risks related to alcohol consumption among Australian men and women. The survey found that 54 % of men and 68 % of women had the perception that they could drink one to two standard drinks every day for many years without adversely affecting their health. Recent evidence shows that even relatively low levels of alcohol consumption are associated with the development of a range of soft tissue cancers [3]. As we have previously noted [11], this evidence essentially changes the known cancer risk status for a significant proportion of the Australian population, to now include those who drink at light-to-moderate levels.

That a substantial proportion of light-to-moderate drinkers may be unknowingly placing themselves at risk has underscored the need for strategies to inform the public of the cancer risk associated with alcohol [12]. Mandated health warnings on alcohol products have been proposed as a cost-effective way to convey this information, which could be worthwhile if part of a multi-pronged public health initiative [13, 14]. Although evaluations of the warning label approach have demonstrated positive effects on knowledge and attitudes [7], there is limited research on how aspects of message content and audience characteristics may influence public perceptions about the effectiveness of cancer-specific warning labels. Furthermore, despite studies reporting support for the introduction of mandated warnings on alcoholic products in Australia [15], little information is available to guide the development of effective warning messages.

Informed by qualitative findings from a series of focus groups in South Australians who self-identified as ‘light-to-moderate’ drinkers, we developed a national online survey to investigate the impact of cancer warning messages on alcohol products and to inform efforts to provide information regarding alcohol-related cancer risk in the Australian context.

Unique to this study is the inclusion of Australian drinkers not ordinarily considered at risk, namely, light-to-moderate drinkers; and the consideration of health messages addressing the long-term, rather than the short-term, harms associated with alcohol.

## Method

Our national online survey was developed using Qualtrics® survey software and was open for a 1-month period in mid-2014 (30th July to 2nd August). The anonymous survey was open to all Australian residents aged 18 years and over, regardless of alcohol consumption. The survey was promoted on Facebook (filtered to exclude members aged under 18 years and those not based in Australia), in community newspapers in all Australian capital cities and was also available on the web pages of a number of community based organisations and government agencies. Representativeness of the sample was not assumed as the method of recruitment was by self-selection. For this reason, we included a range of demographic data in the survey and aimed to recruit a sufficiently large population as to allow for meaningful stratified analysis according to these characteristics. The entry page to the survey included detailed information about the survey including the study aims, potential risks and expected benefits. All individuals were required to consent to participation by acknowledging their understanding of the information provided before the survey could open. By leaving their contact details via a separate link provided at the end of the survey, participants were able to enter a lottery to win one of three $100 shopping vouchers.

### The survey

The survey collected demographic data and information about participants’ usual patterns of drinking behaviour, and included the 3-item Alcohol Use Disorder Identification Test (AUDIT-C), which provides a total score out of 12 across three categories of drinking frequency and quantity [16] and has been found to perform well in general population surveys and among young adults [17]. We applied the widely used cut off score of 4 and above to indicate for problematic alcohol use in both men and women. A cut off score of 4 is able to identify hazardous drinking in men with a sensitivity of 0.86 and a specificity of 0.72 [18] with a lower sensitivity of 0.48 but very high specificity of 0.99 in women [19].

In addition to questions concerning attitudes towards warning labels in general, the survey contained specific questions on the proposed warning messages. Four warning messages were included, graphically presented as labels on three different types of alcohol bottles representing wine, spirits and beer. The four cancer warning statements presented on the labels are provided in Table 1. For each of the four labels, participants were asked to rate their level of agreement (on a Likert scale ranging from *“strongly agree”* to 5 *“strongly disagree”*) with impact statements about raising awareness, prompting conversation, influencing drinking behaviour and educating others about cancer risk. The full impact statements are also presented in Table 1.

**Table 1:** Alcohol warning labels statements

Label warning statements^a^	
Label 1	Three drinks a day increases your chance of bowel cancer by 20 %
Label2	Alcohol causes cancer
Label 3	Two or more drinks a day can increase your risk of mouth and throat cancer by over 50 %
Label 4	1 in 5 breast cancers are caused by alcohol
Impact statements	
IS 1	Raise awareness about the link between regular alcohol consumption and cancer
IS 2	Prompt conversations about the cancer risk involved in drinking alcohol regularly
IS 3	Prompt me to drink alcohol less often
IS 4	Prompt my friends to drink alcohol less often
IS 5	Prompt me to talk to my family and/or friends about the cancer risk associated with alcohol
IS 6	Prompt me to educate my children about the cancer risk associated with alcohol

^a^IARC, World Cancer Report [39]; Fedirko et al. [40]; Tramacere et al. [41]; Allen et al. [4] Clarke et al. [42]

Participants were also asked their level of agreement with a series of statements about responses to warning labels in general. These included *‘I always read product labels when I see them’*; *‘There are too many product warning labels, I tend to ignore them’*; *‘Most product warning labels aren’t relevant to me’*; *‘I prefer to have product warning labels so I know what all the risks are’*; and *‘I usually reassess my behaviour according to the product warning label.’*

### Data analysis

In our analyses, Likert scale agreement categories were dichotomised to *“agree”* and *“disagree”* (excluding the *“neither agree nor disagree”* responses) for each statement and label. The variables were further condensed to create unanimous label agreement and disagreement categories for each statement. For instance, the new outcome variable for agreeing the labels would raise awareness contained only those agreeing or disagreeing with on this statement about all four labels. These agreement outcomes were then compared to all demographic variables and responses to general warning labels and AUDIT-C score. As AUDIT scores (range 1 to 12) were not normally distributed, they were dichotomised at the threshold of four, which is the considered the level indicative of potential alcohol disorders [9, 16]. Age was also not normally distributed in our sample and recoding the data into ordinal age categories did not make it possible to demonstrate differences (using Chi-Square and Cramer’s V). For this reason, age was dichotomised to above and below the median as an appropriate cut off.

We developed agreement ratios with confidence intervals using univariate and multivariate techniques where appropriate. Age and sex were included in all multivariate models to control for potential confounding and to control for over representation in the sample. Missing data were excluded from the analyses given the relative completeness of the responses. Data were analysed using Stata (release 13, Stata Corporation, College Station, TX, USA). This project was approved by The University of Adelaide Human Research Ethics Committee.

## Results

One thousand, five hundred and forty-seven people completed the survey and their characteristics are presented in Table 2. The median age of study participants was 43 years and 72 % were female. Seventy-nine per-cent of survey respondents were born in Australia and 2 % identified as Indigenous Australian. The most common fields of employment or study reported by participants were education and training, health, social welfare and retail. Only 2 % of participants identified ‘Alcohol Production and Distribution’ as a main area of work or study (35/1547) but when these were combined with hotel and hospitality workers, approximately 15 % reported working in an ‘alcohol-related industry’. The majority (78 %) of survey respondents reported a tertiary education, which was a higher proportion than in the Australian population aged 25–64 (24 %) [20]. The majority of participants (91 %) identified as current drinkers and, of these, 56 % scored four or above on the AUDIT-C, placing them within the ‘high risk’ range of scores on this measure.

**Table 2:** Participant characteristics (*N* = 1545)

	Male *n* = 435	Female *n* = 1110	Total *n* = 1545
Age – median years (IQ range)	44 (31–56)	43 (30–53)	43 (30–54)
*[Missing data]*	*[Nil]*	*[Nil]*	*[Nil]*
Born in Australia	338 (78 %)	887 (80 %)	1110 (72 %)
*[Missing data]*	*[2 (1 %)]*	*[Nil]*	*[2 (0 %)]*
Indigenous Australian^a^	12 (3 %)	14 (2 %)	26 (2 %)
Tertiary educated	312 (72 %)	892 (81 %)	1204 (78 %)
*[Missing data]*	*[3 (1 %)]*	*[4 (0 %)]*	*[7 (1 %)]*
Common employment fields:			
Education and training	117 (27 %)	421 (38 %)	538 (35 %)
Health	48 (16 %)	256 (23 %)	304 (20 %)
Social Welfare	38 (9 %)	173 (16 %)	211 (14 %)
Retail	50 (12 %)	156 (14 %)	206 (13 %)
In alcohol-related industry^b^	65 (15 %)	164 (15 %)	229 (15 %)
*[Missing data]*	*[Nil]*	*[Nil]*	*[Nil]*
Current alcohol consumption	393 (92 %)	1006 (91 %)	1399 (91 %)
*[Missing data]*	*[9 (2 %)]*	*[7 (1 %)]*	*[16 (1 %)]*
AUDIT-C score ≥ 4^c^	288 (74 %)	491 (49 %)	779 (56 %)
*[Missing data]*	*[4 (1 %)]*	*[5 (1 %)]*	*[9 (1 %)]*

Note: missing data excluded from all analyses

^a^Aboriginal or Torres Strait Islander

^b^Includes those working in alcohol production/distribution, hotels and hospitality

^c^Proportion of current drinkers only

**Fig. 1:**
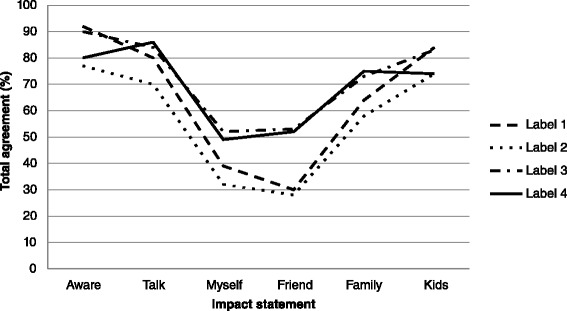
Agreement with impact statements for all four labels. Note. Impact statements are abbreviated to the following: Aware = “Raise awareness about the link between regular alcohol consumption and cancer”. Talk = “Prompt conversations about the cancer risk involved in drinking alcohol regularly”. Myself = “Prompt me to drink alcohol less often”. Friend = “Prompt my friends to drink alcohol less often”. Family = “Prompt me to talk to my family and/or friends about the cancer risks associated with alcohol”. Kids = “Prompt me to educate my children about the cancer risk associated with alcohol”

Overall, the labels were well received, with the majority of respondents (>77 %) agreeing that all four labels could raise awareness about the link between regular alcohol consumption and cancer. There was also majority agreement that the labels would prompt conversations about the cancer risk involved in drinking regularly (>70 %) and prompt participants to educate their children about the cancer risk associated with alcohol (>74 %). There was greater agreement across all impact statements for messages referring to a specific kind of cancer (Labels 1, 3 and 4) than for the message referring to cancer in general (Label 2). More than 80 % of respondents agreed that the three cancer-specific labels would raise awareness of and prompt conversation about the link between alcohol consumption and cancer. About 50 % of respondents or fewer agreed that the labels could influence drinking behaviour, but larger proportions (>58 %) agreed that the labels would prompt them to discuss the issue with family and friends. Figure 1 presents the proportion of total statement agreement (‘agree’ and ‘strongly agree’ combined) for all four labels.

As indicated in Fig. 1, there were similar patterns of response to the four separate labels, therefore unanimous agreement or disagreement to the respective impact statements in all labels was combined. The median age of this subgroup was similar to the total sample (44 years, interquartile range 31–54) as was the proportion of participants scoring ≥ 4 on the Audit-C (53 %). The proportion of females was higher than the total sample (84 % versus 72 %), which was controlled for in the following analyses.

There was 93 % (825/883) total agreement that the labels would raise awareness about the link between alcohol and cancer. There was 86 % (624/724) total agreement that the labels would prompt conversation about the cancer risk involved in drinking regularly and 84 % (616/734) total agreement that they would prompt respondents to educate their children about the cancer risk associated with alcohol consumption. Fewer participants agreed that the labels could influence drinking behaviour. There was 36 % (195/538) total agreement that the labels would prompt the participants themselves to drink alcohol less often, and 30 % (133/446) total agreement that the labels would prompt participants’ friends to drink alcohol less often.

Factors that were associated with agreement with each impact statement are presented in Table 3. There were significant univariate associations between being female and agreement with all six impact statements. For each impact statement, a low AUDIT-C score (<4) was also significantly associated with agreement. The participants who scored as ‘low-risk’ drinkers (i.e. received an overall AUDIT-C score < 4) expressed more agreement with the impact statements than the participants who identified as ‘high-risk’ drinkers. Those aged 43 years (median age) and above were more likely than younger participants to agree that the labels would influence the drinking behaviour of their friends or would prompt them to discuss alcohol and cancer with their friends and/or family. Finally, participants who reported working in an alcohol-related industry (i.e. alcohol production/distribution, hotels or hospitality) were less likely than those working in other areas to agree that the labels would prompt them to talk to family and/or friends, or to educate their children about the cancer risk associated with alcohol.

**Table 3:** Factors univariately associated with total impact statement agreement (*n* = 883)

Statement	Factor	^a^Agreement ratio (95 % CI)	Agreement difference (95 % CI)	*X* ^2^	*p*-value*
Raise awareness about the link between regular alcohol consumption and cancer	Female sex	1.12 (1.06, 1.19)	0.10 (0.06, 0.15)	29.44	<0.001
	AUDIT-C <4	1.05 (1.1, 1.09)	0.04 (0.01, 0.08)	5.48	0.020
	Reassess	1.35 (1.23, 1.48)	0.26 (0.19, 0.32)	114.41	<0.001
	Read	1.22 (1.14, 1.31)	0.18 (0.12, 0.24)	82.36	<0.001
	Ignore	0.77 (0.71, 0.84)	−0.22 (−0.29, −0.16)	111.15	<0.001
	Irrelevant	0.89 (0.84, 0.94)	−0.11 (−0.16, −0.06)	29.84	<0.001
	Risk	1.33 (1.22, 1.45)	0.24 (0.18, 0.31)	137.66	<0.001
Prompt conversations about the cancer risk involved in drinking alcohol regularly	Female sex	1.30 (1.18, 1.43)	0.21 (0.14, 0.28)	50.68	<0.001
	AUDIT-C <4	1.13 (1.06, 1.20)	0.10 (0.05, 0.16)	14.09	<0.001
	Reassess	1.89 (1.62, 2.20)	0.46 (0.37, 0.54)	175.25	<0.001
	Read	1.50 (1.34, 2.67)	0.31 (0.24, 0.39)	114.82	<0.001
	Ignore	0.65 (0.57, 0.74)	−0.33 (−0.41, −0.25)	109.65	<0.001
	Irrelevant	0.78 (0.71, 0.86)	−0.20 (−0.27, −0.13)	46.19	<0.001
	Risk	1.84 (1.59, 2.14)	0.44 (0.36, 0.52)	203.22	<0.001
Prompt me to drink alcohol less often	Female sex	1.61 (1.22, 2.12)	0.16 (0.08, 0.24)	12.93	<0.001
	AUDIT-C <4	1.28 (1.03, 1.60)	0.09 (0.01, 0.17)	4.70	0.030
	Reassess	8.32 (5.00, 13.91)	0.55 (0.48, 0.63)	129.13	<0.001
	Read	1.66 (1.26, 2.19)	0.17 (0.09, 0.25)	14.74	<0.001
	Ignore	0.33 (0.23, 0.49)	−0.30 (−0.38, −0.23)	45.00	<0.001
	Irrelevant	0.48 (0.35, 0.66)	−0.22 (−0.30, −0.15)	25.16	<0.001
	Risk	5.11 (3.33, 7.84)	0.41 (0.35, 0.48)	92.55	<0.001
Prompt my friends to drink alcohol less often	Female sex	1.68 (1.19, 2.36)	0.14 (0.06, 0.22)	9.70	0.002
	AUDIT-C <4	2.10 (1.53, 2.89)	0.21 (0.12, 0.30)	21.35	<0.001
	Age ≥43 years	1.36 (1.02, 1.81)	0.09 (0.01, 0.18)	4.45	0.035
	Reassess	11.58 (6.06, 22.12)	0.56 (0.48, 0.64)	119.66	<0.001
	Read	3.11 (2.02, 4.81)	0.27 (0.19, 0.34)	34.61	<0.001
	Ignore	0.28 (0.18, 0.45)	−0.38 (−0.36, −0.21)	38.05	<0.001
	Irrelevant	0.40 (0.27, 0.60)	−0.23 (−0.31, −0.15)	25.54	<0.001
	Risk	12.06 (5.77, 25.21)	0.43 (0.37, 0.50)	95.70	<0.001
Prompt me to talk to my family and/or friends about the cancer risks associated with alcohol	Female sex	1.61 (1.38, 1.88)	0.30 (0.21, 0.38)	54.77	<0.001
	AUDIT-C <4	1.34 (1.20, 1.50)	0.20 (0.13, 0.28)	26.54	<0.001
	Age ≥43 years	1.17 (1.05, 1.29)	0.11 (0.03, 0.18)	8.53	0.004
	Not working in Alcohol related Industry^b^	1.21 (1.02, 1.45)	0.13 (0.02, 0.23)	6.19	0.013
	Reassess	3.51 (2.71, 4.56)	0.64 (0.57, 0.72)	210.59	<0.001
	Read	1.66 (1.41, 1.94)	0.31 (0.23, 0.39)	60.71	<0.001
	Ignore	0.54 (0.43, 0.64)	−0.45 (−0.37, −0.29)	79.32	<0.001
	Irrelevant	0.66 (0.67, 0.77)	−0.26 (−0.34, −0.18)	41.94	<0.001
	Risk	3.51 (2.69, 4.58)	0.61 (0.54, 0.68)	223.18	<0.001
Prompt me to educate my children about the cancer risk associated with alcohol	Female sex	1.41 (1.27, 1.57)	0.27 (0.2, 0.34)	76.87	<0.001
	Audit-C <4	1.23 (1.15, 1.32)	0.17 (0.12, 0.23)	35.31	<0.001
	Reassess	2.15 (1.81, 2.56)	0.52 (0.44, 0.60)	204.60	<0.001
	Read	1.47 (1.32, 1.64)	0.29 (0.22, 0.37)	92.78	<0.001
	Ignore	0.60 (0.52, 0.69)	−0.37 (−0.45, −0.29)	127.14	<0.001
	Irrelevant	0.74 (0.66, 0.82)	−0.23 (−0.31, −0.16)	55.97	<0.001
	Risk	2.19 (1.84, 2.62)	0.52 (0.44, 0.60)	248.70	<0.001

Notes. Agreement with: generally reassess behaviour based on warning labels is abbreviated to ‘Reassess’; always read product warning labels abbreviated to ‘Read’; too many product warning labels so I tend to ignore them abbreviated to ‘Ignore’; most product warning labels are not relevant to me abbreviated to ‘Irrelevant’; prefer to have product labels so I know what the risks are abbreviated to ‘Risk’

*Chi-Square, 2-tailed tests used

^a^Agreed with impact statement relative to disagreed with impact statement

^b^Includes those working in alcohol production/distribution, hotels and hospitality

A series of Chi square tests were conducted for the association between agreement with the impact statements and preference for warning labels in general (e.g. “*I always read product warning labels when I see them”*). Agreement with the impact statements was significantly associated with attitudes towards warning labels in general (all analyses yielded *P*<0 .001), which included being inclined to read warning labels and reassess behaviour based on their recommendations, and preferring to be informed about risks. Disagreement with the impact statements was associated with the tendency to ignore warning labels in general or perceiving most labels as being personally irrelevant (see Table 3).

In additional analyses, participants aged 43 years and above, low-risk drinkers (AUDIT-C score <4) and females were most likely to report that they always read warning labels, preferred knowing risks and would usually reassess their behaviour according to product warnings. Males, high-risk drinkers and participants aged less than 43 years were least likely to agree with the statement *“There are too many warning product warning labels, I tend to ignore them”*. Being female and median age or above were also significantly associated with having a low-risk AUDIT-C score.

### Multivariate analyses

We built log binomial models to find independent predictors of agreement with each label impact statement. Collinearity between two potential explanatory variables was assessed using Chi-square and Phi. All models included age and sex and various combinations of AUDIT-C score, working in an alcohol-related industry and education level, as well as reported responses to warning labels in general. After adjusting for sex, a low AUDIT-C score was no longer associated with agreement that the labels would raise awareness about the cancer risk involved in consuming alcohol regularly or prompt participants to drink alcohol less often. Neither education level nor working in the alcohol industry significantly predicted agreement with any of the impact statements after adjustment for sex, age and AUDIT-C score.

In models including participants’ preference to read warning labels in general, being female continued to be a significant predictor of statement agreement on five of the six impact statements. Being female was no longer associated with participant agreement that the labels would prompt them to drink alcohol less often, once adjusted for preference to read warning labels in general. Despite the significant univariate associations observed, neither age nor AUDIT-C score were significant predictors of agreement that the labels would raise awareness and prompt conversation about the cancer risk involved in consuming alcohol regularly, prompt participants to drink less, or prompt them to educate their children about the cancer risk associated with drinking alcohol, after adjustment for sex and preference to read warning labels.

AUDIT-C score and age did not predict agreement with any of the impact statements after adjusting for sex and reassessing behaviour based on warning labels. When preference to read warning labels was included, our models indicated that being female, reading labels and reassessing behaviour based on general product warnings independently predicted agreement that the alcohol labels would raise awareness of and prompt conversation about the cancer risk associated with alcohol consumption, prompt participants themselves to drink alcohol less often and prompt participants to educate their children about the alcohol-cancer link. However, “*I always read product labels when I see them”* and *“I usually reassess my behaviour according to the product warning label”* were found to be significantly associated with each other, with 90 % agreement (Chi-2 = 271.8, *p* < 0.001) and were also strongly correlated (Phi = 0.522). Convergence was difficult to reach when both were included in the most models due to this collinearity. For this reason, these factors were included in a series of separate multivariate models with “*I usually reassess my behaviour according to the product warning label*” emerging as the more important factor.

Further investigations of the interrelationships between the attitudes to warning labels in general it emerged that ‘*tend to ignore product warning labels*’ and ‘*most product warning labels are not relevant to me*’ were no longer significant for any impact statement once controlling for ‘*usually reassessing behaviour*.’ Significant collinearity was also found between “*I prefer to have product labels so I know all of the risks”* and *“I usually reassess my behaviour according to the product warning label”* with 97 % agreement (Chi-2 = 487.15, *p* < 0.001) and were also strongly correlated (Phi = 0.700). The models including the stronger factor are presented in Table 4, as based on size of the ratio and reduction of the significance of other factors in the model.

**Table 4:** Factors predicting statement agreement: multivariate analysis

Statement	Factor	Agreement ratio (95 % CI)	Agreement difference (95 % CI)	*p*-value
Raise awareness about the link between regular alcohol consumption and cancer	Female sex	1.07 (1.04–1.10)	0.04 (0.01–0.07)	<0.001
	Risk	1.32 (1.22–1.44)	0.24 (0.17–0.30)	<0.001
Prompt conversations about the cancer risk involved in drinking alcohol regularly	Reassess	1.87 (1.61–2.18)	0.45 (0.36–0.53)	<0.001
Prompt me to drink alcohol less often	Reassess	8.59 (5.13–14.40)	0.56 (0.48–0.53)	<0.001
Prompt my friends to drink alcohol less often	≥43 years	1.40 (1.05–1.86)	0.09 (0.04–0.14)	0.024
	Risk	9.45 (4.50–19.90)	0.37 (0.30–0.46)	<0.001
	AUDIT-C <4	1.42 (1.06–1.89)	0.11 (0.02–0.16)	0.019
Prompt me to talk to my family/friends about the cancer risks associated with alcohol	Reassess	3.44 (2.61–4.54)	0.52 (0.22–0.63)	<0.001
Prompt me to educate my children about the cancer risk associated with alcohol	Reassess	14.71 (8.16–26.51)	0.49 (0.41–0.58)	<0.001

Notes. Agreement that with the statement about generally reassessing behaviour based on warning labels is abbreviated to ‘Reassess’; prefer to have product labels so I know what the risks are abbreviated to ‘Risk’

All models included age and sex

Our final models are presented in Table 4, and include the remaining significant factors after adjusting for age and sex. These analyses indicated that positive responses to warning labels in general was the single most important predictor of agreement with all of the impact statements. Being female continued to predict agreement that the labels would raise awareness and prompt behaviour changes in friends, while having a low AUDIT-C score predicted agreement that the labels might prompt behaviour change in friends.

## Discussion

This study aimed to canvass responses of the Australian public to cancer warning labels on alcohol products. Several studies have demonstrated that there is substantial public support for the introduction of alcohol warning labels in Australia [15, 21, 22]. Our findings suggest that cancer warning statements are unlikely to be received negatively by the Australian community, with the majority of participants agreeing that all labels could raise awareness of, and prompt conversations about, the cancer risk associated with alcohol. Similar outcomes were observed in a recent Australian study, which reported that responses to cancer-related warning labels were generally neutral to positive [7].

Our results yielded significant differences in the outcome variables by message and respondent characteristics. Females were more likely to report usually acting upon the advice of warning labels, with the latter characteristics independently associated with agreement that the labels raise awareness and prompt behaviour changes. There was higher agreement across all impact statements for messages referring to a specific type of cancer than for the message referring to cancer in general. This finding is consistent with those of Pettigrew et al. [8], where participants reported that specific cancer warnings were more believable, convincing and personally relevant than general cancer warnings.

Although there was broad agreement about the capacity of the labels to raise awareness and prompt discussion about alcohol and cancer, it is uncertain whether any improvements to knowledge resulting from exposure to the labels will elicit sustained behaviour change. Reviews of the literature suggest that alcohol warning messages may improve knowledge and attitudes relating to the harmful consequences of alcohol use in adults [17]; however, there is limited evidence of the effects of these messages on drinking behaviour [13]. In the present study, less than half of all respondents agreed that the warning messages could influence their own drinking behaviour and even fewer agreed the messages might influence the drinking behaviour of their friends. Similar results have been reported in studies conducted in the United States, where warning labels about the adverse effects of alcohol on pregnancy, driving ability and health have been mandatory since November 1989 [17]. In one example, Greenfield et al. [23] reported that exposure to a warning label was associated with modest effects on discussing alcohol-related risks, and small effects on precautionary behaviours related to the risk of drinking.

Similar findings have been reported in adolescent samples. A review of the literature investigating the impact of alcohol labelling on adolescents’ drinking knowledge and behaviour found that the introduction of warning labels was associated with improved awareness and recognition of the warning messages, despite little change in actual behaviour [21]. Since attitudes regarding alcohol consumption are often formed during adolescence [24] and carcinogenesis is a generally a slow process [25], dealing with perceptions, attitudes and drinking behaviour during this period of development is critical.

Our univariate findings suggested that being female and self-reported low-risk drinking status were factors associated with agreement with the impact statements. However, a significantly higher proportion of males than females in this study were identified as high-risk drinkers based on their overall AUDIT-C scores (74 % versus 49 %). This pattern is consistent with previous Australian studies that have reported higher levels of harmful alcohol consumption among males than females [26]. Despite the significant univariate associations observed, final multivariate models indicated that the most significant predictor of agreement with the impact statements was a high level of responsiveness to warning labels in general. The results indicated that participants with this characteristics were also more likely to drink at low-risk levels. These findings are supported by previous research, which has demonstrated a negative association between consumption of alcohol and the behaviour of reading product warning labels [27].

Participants who reported drinking at high-risk levels were less likely to read product warnings in general or comply with their recommendations than participants who reported drinking at low-risk levels. DeCarlo [27] has explained this relationship from the perspective of social judgement theory, which states that lower-involvement individuals (such as light drinkers) are more likely to pay attention to and accept persuasive efforts than high-involvement individuals (such as heavy drinkers). According to the theory, behavioural change resulting from persuasive messages is constrained by the importance and frequency of the behaviour [25, 28]. On this basis, a behaviour that is considered important or is frequently performed will be resistant to change [25]. This contrast effect was evident in a study by Bozinoff et al. [29] in which the effectiveness of alcohol awareness campaigns was significantly greater for non-drinkers than for heavy drinkers of alcohol. Alternatively, negative responses to specific alcohol warnings in heavier drinkers could be attributed to psychological reactance, in which the threat to freedom might paradoxically motivate increased alcohol consumption [30, 31].

Our results indicated that high-risk drinkers perceived the labels to be less effective in altering drinking behaviour than light-to-moderate drinkers, and reported they would be less likely to modify their behaviour based on health warnings or read warning labels in general. This suggests that alcohol warning label information may be disregarded by those who are most at risk of harmful drinking, which is consistent with previous research [27, 32, 33]. However, in light of recent evidence demonstrating an association between regular, low levels of alcohol consumption and several types of cancer [34] even light-to-moderate drinkers are at risk of developing an alcohol-related cancer. Consequently, although cancer warnings may have less impact on heavier drinkers, they may positively influence the drinking behaviours of those who consume alcohol at more moderate levels and are still at risk for cancer.

As the survey was open to all Australian adults 18 years of age and older, the sample differed from the general adult population in a number of ways. Despite employing a broad range of general and targeted recruitment strategies, our respondents were older, more likely to be female and more likely to be university educated than the general Australian population [35]. Although the number of respondents made it possible to control for these differences in our analyses, it is important to consider representativeness when generalising our findings to the whole population.

Participants in the study were exposed to four warning labels presented in the same order, which had the potential to affect overall responses. However, that we observed a similar pattern of reduced impact agreement for the simple ‘Alcohol causes cancer’ message that appeared second (sandwiched between labels with detailed messages), provides some confidence that responses were not necessarily dependent on order of label presentation. Further study is required to determine whether the responses would be consistent when such labels were encountered on alcohol products in a social setting.

Our study focused on label messages rather than other aspects that may have an influence on acceptance and impact. Future studies of the broad population might explore the impact of label colour, placement and origin of the message, including messages originating from the government and from key community organisations. Laughery et al. [36] experimented with various levels of explicitness in warning message and determined that explicit messages were likely to be more effective than vague warnings and, as mentioned, the more detailed messages were received more positively by our participants. The authors identified many characteristics as key for effectively communicating explicit product warning labels and improving noticeability, including many of the characteristics listed above.

Further research is required to determine if and how the cancer information conveyed by warning labels is retained. In a study that examined the impact of warning labels up to 5 years after their introduction, it was found that the observed positive effects on awareness and recognition were not maintained over time [37]. Future studies would therefore benefit from the inclusion of follow-up assessments, to discern the long-term effectiveness of the warning label approach. The public health consequences of potential paradoxical responses due to psychological reactance [31] should also not be overlooked in future investigations of long-term effectiveness. One other issue for future research may be to correlate the desire to reduce drinking with the effectiveness of alcohol labels.

As Louise et al. [12] discuss, the alcohol warning label approach does present ethical issues around relying on generating fear in relation to cancer in order to impact on behaviour. Yet the effective potential effectiveness of alcohol warning labels may actually *depend* on the emotion of fear rather than simply communicating the health promoting message on the link between alcohol and cancer. Generally, the successful ‘fear appeal’ message will contain three parts – the emotion of fear, the cognition of threat (i.e. perceived susceptibility), and the perception of self-efficacy to control and respond adequately to the threat [38]. For alcohol warning labels to be effective, it is likely that other methods focussing on all three aspects will be required.

## Conclusion

Although alcohol labels may raise awareness of and prompt discussions about the messages they contain, the wider literature and our own findings suggest they might produce only limited effects on drinking behaviour on their own. To maximise impact, warning labels should be considered for use in conjunction with other avenues for prevention, and incorporated into multi-faceted health campaigns.

## References

[1] Australian Institute of Health and Welfare (2011). Drugs in Australia 2010: tobacco, alcohol and other drugs. Drug statistics series no. 27.

[2] International Agency for Research on Cancer. IARC monographs on the evaluation of carcinogenic risks to humans, volume 96: Alcohol consumption and ethyl carbonate. World Health Organization / International Agency for Research on Cancer. 2014. http://monographs.iarc.fr/ENG/Monographs/vol96/mono96.pdf. Accessed 31 Mar 2015.

[3] Winstanley MH, Pratt IS, Chapman K, Griffin HJ, Croager EJ, Olver IN (2011). Alcohol and cancer: a position statement from Cancer Council Australia. Med J Aust.

[4] Allen NE, Beral V, Casabonne D, Kan SW, Reeves GK, Brown A (2009). Moderate alcohol intake and cancer incidence in women. J Natl Cancer Inst.

[5] Corrao G, Bagnardi V, Zambon A, La Vecchia C (2004). A meta-analysis of alcohol consumption and the risk of 15 diseases. Prev Med.

[6] American Institute for Cancer Research. Policy and action for cancer prevention. Food, nutrition, and physical activity: a global perspective. World Cancer Research Fund / American Institute for Cancer Research. 2009. http://www.aicr.org/assets/docs/pdf/reports/Second_Expert_Report.pdf. Accessed 31 Mar 2015.

[7] Stewart B, Wild C, eds. World Cancer Report 2014. International Agency for Research on Cancer / World Health Organization. Lyon; 2014.

[8] Pettigrew S, Jongenelis M, Chikritzhs T, Slevin T, Pratt IS, Glance D (2014). Developing cancer warning statements for alcoholic beverages. BMC Public Health.

[9] National Health Medical Research Council (2009). Australian guidelines to reduce health risks from drinking alcohol.

[10] Australian Institute of Health and Welfare (2014). National Drug Strategy Household Survey detailed report 2013. Drug statistics series no. 28.

[11] Eliott JA, Miller ER (2014). Alcohol and cancer: the urgent need for a new message. Med J Aust.

[12] Louise J, Eliott J, Olver I, Braunack-Mayer A (2015) Mandatory cancer risk warnings on alcoholic beverages: What are the ethical issues? The American Journal of Bioethics, 15(3): 3–11, 2015. Am J Botethics 15(3): 3–11, 2015 (3):3–11 10.1080/15265161.2014.998373 25786002

[13] Stockwell T (2006). A review of research into the impacts of alcohol warning labels on attitudes and behaviour.

[14] Cancer Council Australia (2013). Position statement: Consumer information and labelling of alcohol.

[15] Thomson LM, Vandenberg B, Fitzgerald JL (2012). An exploratory study of drinkers views of health information and warning labels on alcohol containers. Drug Alcohol Rev.

[16] Aalto M, Alho H, Halme JT, Seppä K (2009). AUDIT and its abbreviated versions in detecting heavy and binge drinking in a general population survey. Drug Alcohol Depend.

[17] Kelly TM, Donovan JE, Chung T, Bukstein OG, Cornelius JR (2009). Brief screens for detecting alcohol use disorder among 18–20 year old young adults in emergency departments: Comparing AUDIT-C, CRAFFT, RAPS4-QF, FAST, RUFT-Cut, and DSM-IV 2-Item Scale. Addict Behav.

[18] Bush K, Kivlaham DR, McDonell MB, Fihn SD, Gradley KA, for the Ambulatory Care Quality Improvement Project (AcQUIP) (1998). The AUDIT Alcohol Consumption Questions (AUDIT-C) an effective brief screening test for problem drinking. Arch Intern Med.

[19] Bradley KA, Bush KR, Epler AJ, Dobie DJ, Davis TM, Sporleder JL (2003). Two brief Alcohol-screening tests from the Alcohol Use Disorders Identification Test (AUDIT). Arch Intern Med.

[20] Australian Bureau of Statistics (2014). Population by Age and Sex, Regions of Australia.

[21] Scholes-Balog KE, Heerde JA, Hemphill SA (2012). Alcohol warning labels: Unlikely to affect alcohol-related beliefs and behaviours in adolescents. Aust N Z J Public Health.

[22] Wilkinson C, Room R (2009). Warnings on alcohol containers and advertisements: international experience and evidence on effects. Drug Alcohol Rev.

[23] Greenfield TK, Ye Y, Giesbrecht NA (2007). Views of alcohol control policies in the 2000 National Alcohol Survey: What news for alcohol policy development in the US and its States?. J Subst Use.

[24] Newcomb MD, Maddahian E, Bentler PM (1986). Risk factors for drug use among adolescents: concurrent and longitudinal analyses. Am J Public Health.

[25] Cuzick J (2010). Long-term follow-up in cancer prevention trials (it ain’t over’til it’s over). Cancer Prev Res.

[26] Bowden JA, Delfabbro P, Room R, Miller CL, Wilson C (2014). Alcohol consumption and NHMRC guidelines: has the message got out, are people conforming and are they aware that alcohol causes cancer?. Aust N Z J Public Health.

[27] DeCarlo TE (1997). Alcohol warnings and warning labels: an examination of alternative alcohol warning messages and perceived effectiveness. J Consum Mark.

[28] Wu C, Shaffer DR (1987). Susceptibility to persuasive appeals as a function of source credibility and prior experience with the attitude object. J Pers Soc Psychol.

[29] Bozinoff L, Roth V, May C (1989). Stages of involvement with drugs and alcohol: Analysis of effects of drug and alcohol abuse advertising. Adv Consum Res.

[30] Brown KG, Stautz K, Hollands GJ, Winpenny EM, Marteau TM (2015). The Cognitive and Behavioural Impact of Alcohol Promoting and Alcohol Warning Advertisements: An Experimental Study. Alcohol Alcohol.

[31] Bensley LS, Wu R (1991). The Role of Psychological Reactance in Drinking Following Alcohol Prevention Messages1. J Appl Soc Psychol.

[32] Andrews JC, Netemeyer RG (1996). Alcohol warning label effects: Socialization, addiction, and public policy issues.

[33] Hankin JR, Sloan JJ, Firestone IJ, Ager JW, Sokol RJ, Martier SS (1993). A time series analysis of the impact of the alcohol warning label on antenatal drinking. Alcohol Clin Exp Res.

[34] Australian Institute of Health and Welfare. 2004 National Drug Strategy Household Survey: detailed findings. Australian Institute of Health and Welfare. 2005. http://www.aihw.gov.au/WorkArea/DownloadAsset.aspx?id=6442459722. Accessed 31 Mar 2015

[35] Australian Bureau of Statistics (2014). Population Estimates by Age and Sex, Regions of Australia (ASGS 2011), 2001 to 2013.

[36] Laughery KR, Vaubel KP, Young SL, Brelsford JW, Rowe AL (1993). Explicitness of Consequence Information in Warnings. Saf Sci.

[37] MacKinnon DP, Nohre L, Pentz MA, Stacy AW (2000). The alcohol warning and adolescents: 5-year effects. Am J Public Health.

[38] Witte K, Allen M (2000). A Meta-analysis of fear appeals: implications for effective public health Campaigns. Health Ed Behav.

[39] Stewart B, Kleihues P, eds. IARC, World Cancer Report. IARC. Lyon; 2003.

[40] Fedirko V, Tramacere I, Bagnardi V, Rota M, Scotti L, Islami F (2011). Alcohol drinking and colorectal cancer risk: an overall and dose–response meta-analysis of published studies. Ann Oncol.

[41] Tramacere I, Negri E, Bagnardi V, Garavello W, Rota M, Scotti L (2010). A meta-analysis of alcohol drinking and oral and pharyngeal cancers. Part 1: Overall results and dose-risk relation. Oral Oncol.

[42] Clarke CA, Purdie DM, Glaser SL (2006). Population attributable risk of breast cancer in white women associated with immediately modifiable risk factors. BMC Cancer.

